# Steroid use in PROWESS severe sepsis patients treated with drotrecogin alfa (activated)

**DOI:** 10.1186/cc3778

**Published:** 2005-07-27

**Authors:** Howard Levy, Pierre-Francois Laterre, Becky Bates, Rebecca L Qualy

**Affiliations:** 1 Medical Director, Acute Care, Eli Lilly and Co., Indianapolis, Indiana, USA; 2 Professor in Medicine, Head of Intensive Care Medicine, Critical Care and Emergency Department, Cliniques Universitaires St Luc, UCL, Brussels, Belgium; 3 Associate Senior Statistician, Eli Lilly and Co., Indianapolis, Indiana, USA; 4 Senior Scientific Communications Associate, Eli Lilly and Co., Indianapolis, Indiana, USA

## Abstract

**Introduction:**

In a study conducted by Annane, patients with septic shock and unresponsive to adrenocorticotropic hormone stimulation receiving low-dose steroid therapy had prolonged survival but not significantly improved 28-day mortality. The present study examines intravenous steroid use in PROWESS (Recombinant Human Activated Protein C Worldwide Evaluation in Severe Sepsis) patients meeting the Annane enrollment criteria (AEC).

**Methods:**

Adrenocorticotropic hormone stimulation tests were not done in PROWESS. Steroids were allowed but their use was not directed. Patients were identified using AEC (all of: randomization to study drug treatment within 8 hours of shock onset; infection, fever, or hypothermia; tachycardia; systolic blood pressure <90 mmHg on vasopressors; mechanical ventilation; and one of urine <0.5 ml/kg per hour, lactic acidosis, or arterial oxygen tension/inspired fractional oxygen <280). We examined steroid use and mortality data; additional analyses were done outside the 8-hour window.

**Results:**

Steroid-treated patients were older, had higher Acute Physiology and Chronic Health Evaluation scores and more organ dysfunctions, and were more commonly receiving mechanical ventilation. Among patients meeting AEC, regardless of steroid treatment (*n *= 97), mortality in the placebo and drotrecogin alfa (activated) groups was 38% (19/50) and 28% (13/47), respectively (relative risk [RR] = 0.73, 95% confidence interval [CI] 0.41–1.30). When using AEC but excluding the requirement for randomization within 8 hours of shock onset (*n *= 612), placebo mortality was 38% (118/313) and drotrecogin alfa (activated) mortality was 29% (88/299; RR = 0.78, 95% CI 0.62–0.98). Using AEC but excluding the 8-hour window and with steroids initiated at baseline and/or infusion (*n *= 228) resulted in mortality for placebo and drotrecogin alfa (activated) groups of 43% (51/118) and 33% (36/110), respectively (RR = 0.76, 95% CI 0.54–1.06).

**Conclusion:**

Patients with severe sepsis from the PROWESS trial who were likely to respond to low-dose steroids according to the AEC were those patients at a high risk for death. However, when using the AEC, regardless of steroid use, patients exhibited a survival benefit from treatment with drotrecogin alfa (activated).

## Introduction

Corticosteroid therapy in sepsis and septic shock has been investigated for more than 50 years [1]. Over this period there have been dozens of trials examining various patient populations, assessing different corticosteroids in a wide range of dosing regimens, and employing methodologies that are diverse in form and quality [1-15]. Results have varied widely, with some studies favoring the control group and some favoring the treatment group (low-dose use); others have shown virtually no difference in outcome, and still other studies (particularly those examining high-dose steroids) have indicated that steroid therapy is harmful [3,4,9,16]. Recently, a small study of patients with community-acquired pneumonia [17] showed a positive effect of steroid treatment. However, findings from several investigators suggest that steroid treatment should be limited to patients who have adrenal insufficiency [18-21].

The hypothalamic–pituitary–adrenal axis plays an important role in the body's ability to respond to stress. Patients who develop septic shock and who are consequently maximally stressed, in response to an infection, may exhibit adrenal insufficiency. Insufficiency of the adrenal system correlates with increased risk for mortality associated with severe sepsis and/or septic shock [22]. Adrenal replacement therapy in patients with adrenal failure may be a logical addition to standard care in patients with severe sepsis and vasopressor dependent shock. Annane and colleagues [22] demonstrated that the response to a short corticotropin test could potentially be used to identify patients with relative adrenal insufficiency who are at high risk for death related to septic shock. In another recent, randomized trial of 300 patients with septic shock, Annane and coworkers [15] found that 229 patients (about two-thirds) had adrenal insufficiency, as determined using the 250 μg corticotropin stimulation test. In this subgroup mortality at 28 days was not significantly less among those who received corticosteroids (53%) than in the placebo group (63%; *P *= 0.10). Patients who had adrenal insufficiency appeared to have prolonged median survival (16.5 days for corticosteroid treatment versus 14 days for placebo), but these values and the difference in 28-day mortality between treatment group and placebo were not significant.

A recent review and meta-analysis [18] assessing the effects of corticosteroids on mortality in patients with severe sepsis and septic shock found that, for all published trials, use of corticosteroids did not significantly affect mortality overall. The studies by Annane and coworkers did show that corticosteroid treatment might reduce mortality in a subgroup of septic shock patients with well defined adrenal insufficiency. However, even with corticosteroid plus fludrocortisone treatment, more than half of that subgroup of patients died, clearly indicating the importance of additional therapies to reduce mortality not only in this subgroup but also in the overall population of septic shock patients, with or without adrenal insufficiency.

There are currently no published data on the use of drotrecogin alfa (activated) with corticosteroids in the treatment of severe sepsis. The Recombinant Human Activated Protein C Worldwide Evaluation in Severe Sepsis (PROWESS) trial [23] was a phase III study designed to evaluate drotrecogin alfa (activated) for the treatment of patients with severe sepsis at high risk for death (e.g. as determined by an Acute Physiology and Chronic Health Evaluation II score ≥25 and/or two or more organ dysfunctions). The present study examines steroid use in PROWESS patients with severe sepsis and septic shock.

## Materials and methods

In the PROWESS trial, severe sepsis patients were randomly assigned to receive either drotrecogin alfa (activated) at a dose of 24 μg/kg per hour, or placebo, administered intravenously for 96 hours. Concomitant use of steroids was allowed but was not required or specified by the protocol in PROWESS. The duration and route but not dose of steroids was recorded. For the present analysis, patients were identified using all of the Annane enrollment criteria (AEC): randomization of treatment with drotrecogin alfa (activated) or placebo within 8 hours of onset of shock; infection, fever, or hypothermia; tachycardia; systolic blood pressure <90 mmHg on vasopressors; mechanical ventilation; and one of urine output <0.5 ml/kg per hour, lactic acidosis, or arterial oxygen tension/fractional inspired oxygen <280. In the PROWESS trial patients were classified as being in septic shock at baseline if they met any of the following criteria for at least 1 hour despite adequate fluid resuscitation or having documented adequate intravascular volume status, at any time within the 6 hours before the start of infusion of drotrecogin alfa (activated) or placebo: arterial systolic blood pressure ≤90 mmHg; mean arterial pressure ≤70 mmHg; or need for vasopressors (defined as dopamine ≥5 μg/kg per min or noradrenaline [norepinephrine], adrenaline [epinephrine], or phenylephrine at any dose) to maintain systolic blood pressure ≥90 mmHg or mean arterial pressure ≥70 mmHg.

We also analyzed data from PROWESS patients selected using the AEC but without the criterion of drotrecogin alfa (activated) or placebo treatment initiation within 8 hours of the onset of septic shock. The adrenocorticotropic hormone stimulation test was not done in PROWESS, and so subgroups related to adrenal insufficiency could not be evaluated.

The characteristics of patients receiving steroids at baseline or during infusion were compared with those of patients who did not receive steroids at baseline or during infusion. Continuous baseline characteristics (e.g. age) were analyzed using one-way analysis of variance. Categorical baseline characteristics were analyzed using Pearson's χ^2 ^test.

Pearson's χ^2 ^tests were used for all 28-day mortality subgroup analyses, which compared drotrecogin alfa (activated) treated patients with placebo patients. The logit methodology was used to calculate relative risks and associated 95% confidence intervals.

## Results

**Figure 1:**
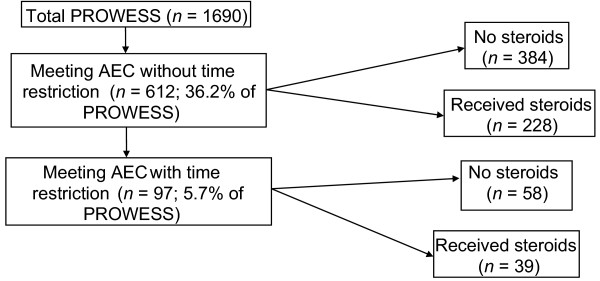
Patient population. AEC = Annane enrollment criteria.

Baseline characteristics (e.g. age, disease severity, etc.) were not different between placebo and drotrecogin alfa (activated) treated patients in the PROWESS trial [23]. The distribution of patients from PROWESS according to AEC is shown in Fig. 1. Of the 1690 PROWESS patients, 36.2% met the AEC without the 8-hour time restriction (i.e. randomization to study drug treatment within 8 hours of shock onset) and 5.7% met the AEC with the 8-hour time restriction. These two groups were then further subdivided into patients receiving steroids and those not receiving steroids. Patients receiving steroid treatment at either severe sepsis onset or during drotrecogin alfa (activated) infusion were classified as treated with steroids.

**Figure 2:**
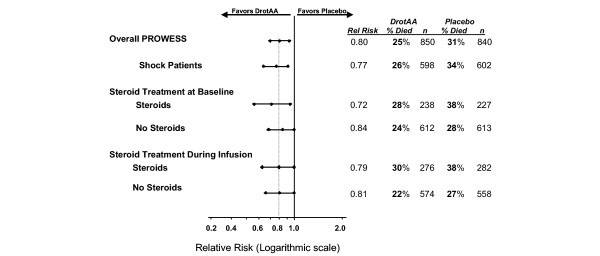
28-Day all-cause mortality for PROWESS patients. Infusion refers to infusion period plus 1 calendar day after termination of infusion. DrotAA, drotrecogin alfa (activated); PROWESS, Recombinant Human Activated Protein C Worldwide Evaluation in Severe Sepsis.

Table 1 lists the baseline disease severity measures for PROWESS patients treated or not treated with steroids (PROWESS overall; AEC not considered). Patients treated with steroids were older, and had higher mean Acute Physiology and Chronic Health Evaluation II scores and more organ dysfunctions than did patients not receiving steroids. Patients were also more likely to receive ventilator support in the steroid treatment group at baseline. PROWESS 28-day all-cause mortality by steroid exposure either at severe sepsis onset or drotrecogin alfa (activated) infusion is shown in Fig. 2.

**Figure 3:**
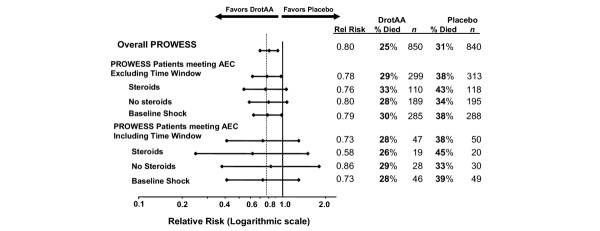
28-Day all-cause mortality for PROWESS Patients meeting the Annane enrollment criteria. Steroids received at baseline and/or during the infusion period. AEC = Annane enrollment criteria; DrotAA, drotrecogin alfa (activated); PROWESS, Recombinant Human Activated Protein C Worldwide Evaluation in Severe Sepsis.

Figure 3 illustrates the 28-day all-cause mortality for PROWESS patients meeting the AEC, either including or excluding the 8-hour time window (study treatment within 8 hours of onset of shock). A survival benefit was observed for drotrecogin alfa (activated)-treated patients regardless of whether they were treated with steroids at baseline or during infusion, or whether they met the AEC with or without the 8-hour time criteria.

## Discussion

The use of steroid therapy in the treatment of sepsis and septic shock has been a controversial issue for many decades. Recent data [15] indicate that physiologic doses of hydrocortisone and fludrocortisone used in combination can reduce the risk for death in patients with relative adrenal insufficiency and septic shock. However, the patient population in that study remained at a high risk for death, as indicated by a 28-day mortality rate of 53% in the treatment group in which steroids were most effective. Guidelines from the Surviving Sepsis Campaign [24] suggested that stress dose steroid therapy should be used for septic shock; however, they further stated that there are no documented studies showing that stress doses of steroids improve the outcome of sepsis without shock unless a patient's history indicates steroid use or adrenal dysfunction. In the recent meta-analysis conducted by Annane and coworkers [18] it was concluded that steroids should be given to patients only when absolute or relative adrenal insufficiency is present. However, the definition for adrenal insufficiency has varied in the few trials in which it was used to evaluate patients for steroid treatment [12,15]. A further area of controversy is whether serum cortisol levels should be measured as total or free cortisol. It was recently reported that severe hypoproteinemia frequently results in concentrations of serum total cortisol in critically ill patients that are lower than expected, whereas free cortisol levels give a more accurate indication of response to corticotropin stimulation [25] and thus provide better identification of patients with adrenal insufficiency.

The PROWESS trial was a phase III placebo-controlled study that evaluated drotrecogin alfa (activated) for the treatment of patients with severe sepsis [23]. In that study drotrecogin alfa (activated) treatment was associated with a significant absolute reduction in mortality rate of 6.1% (relative risk reduction 19.4%; *P *= 0.005), and of 12.8% (relative risk reduction 29.2%; *P *= 0.0002) in the subpopulation of patients who were at high risk for death, which led to its approval by the US Food and Drug Administration.

This is the first report on the use of drotrecogin alfa (activated) with corticosteroids in the treatment of severe sepsis. An analysis of the PROWESS data indicates that 36.2% of the 1690 PROWESS patients met the AEC without the 8-hour time restriction and 5.7% met the criteria with the 8-hour time restriction for enrollment in the study by Annane and coworkers [15]. Limitations of our study include the fact that we did not know the dose or particular type of corticosteroid drug administered and that we did not know the responsiveness of patients to the adrenocorticotropic hormone test.

When examining data from PROWESS, mortality among placebo patients does not differ regardless of whether steroid was given at baseline or during infusion, or whether one applies the 8-hour time restriction or not. Where no steroid was given, the mortality in the two groups still does not differ, suggesting that the timing of steroid treatment alone does not affect mortality. These data further demonstrate an absence of effect of steroid treatment on the potential benefit from drotrecogin alfa (activated) treatment.

The mortality rate from severe sepsis in the PROWESS trial was substantially lower than that previously reported by Annane and coworkers [15,18]. However, the PROWESS trial employed different exclusion criteria than did Annane and coworkers; in particular, the PROWESS trial excluded moribund patients and patients not expected to survive 28 days because of an underlying medical disease.

Drotrecogin alfa (activated) reduced mortality in PROWESS patients with severe sepsis at high risk for death [23]. Patients at high risk for death were more likely to be treated with steroids. In the PROWESS trial the use of steroids did not significantly affect the treatment benefit from drotrecogin alfa (activated).

## Conclusion

Drotrecogin alfa (activated) reduces mortality in patients with severe sepsis at high risk for death, as indicated by meeting the AEC for steroid use. Therefore, we conclude that severe sepsis patients with vasopressor dependent shock should be evaluated for drotrecogin alfa (activated) therapy, particularly if steroids are considered. This is because, regardless of steroid use, these patients have demonstrated survival benefit from treatment with drotrecogin alfa (activated).

## Key messages

• 	Meeting criteria for steroid use by Annane study entry criteria identifies a patient at high risk of death.

• 	Patients receiving steroids in PROWESS were older, had higher APACHE II scores, more organ dysfunctions, and were more commonly receiving mechanical ventilation than those who did not receive steroids.

• 	Drotrecogin alfa (activated) provides a survival benefit to these high-risk patients regardless of steroid use.

## Abbreviations

AEC = Annane enrollment criteria.

## Competing interests

Howard Levy, Becky Bates, and Rebecca L Qualy are employees and shareholders of Eli Lilly and Company. Pierre-Francois Laterre was an investigator in the PROWESS trial, and is a paid consultant and speaker for Eli Lilly and Company.

## Authors' contributions

All the authors contributed to the composition, revision and review of the manuscript, and have read and approved the final version. In addition, HL conceived the idea for this report, BB performed the statistical analysis, RLQ drafted the document and P-FL participated in obtaining the original PROWESS data.

## Figures and Tables

**Table 1:** Baseline disease severity measures for patients treated versus not treated with steroids

Characteristic/parameter	Steroids^a^	No steroids	*P *value
Age (years; mean ± SD [*n*])			
Overall	62 ± 15.9 (586)	60 ± 17.3 (1104)	0.024^b^
Drotrecogin alfa (activated)	62 ± 16.1 (291)	60 ± 17.7 (559)	0.126^b^
Placebo	62 ± 15.7 (295)	60 ± 16.8 (545)	0.098^b^

APACHE II (mean ± SD [*n*])			
Overall	26.4 ± 7.6 (586)	23.9 ± 7.6 (1104)	<0.001^b^
Drotrecogin alfa (activated)	26.6 ± 7.7 (291)	23.5 ± 7.4 (559)	<0.001^b^
Placebo	26.2 ± 7.5 (295)	24.3 ± 7.9 (545)	<0.001^b^

Number of organ dysfunctions (mean ± SD [*n*])			
Overall	2.5 ± 1.1 (586)	2.3 ± 1.1 (1104)	0.018^b^
Drotrecogin alfa (activated)	2.5 ± 1.1 (291)	2.3 ± 1.1 (559)	0.020^b^
Placebo	2.5 ± 1.1 (295)	2.4 ± 1.1 (545)	0.301^b^

Baseline shock^d ^(% [*n*])			
Overall	74% (432)	70% (768)	0.073^c^
Drotrecogin alfa (activated)	75% (217)	68% (381)	0.521^c^
Placebo	73% (215)	71% (387)	0.565^c^

Baseline vasopressor (% [*n*])			
Overall	64% (374)	62% (683)	0.429^c^
Drotrecogin alfa (activated)	62% (179)	60% (337)	0.728^c^
Placebo	66% (195)	63% (346)	0.450^c^

Baseline ventilator (% [*n*])			
Overall	80% (471)	73% (804)	<0.001^c^
Drotrecogin alfa (activated)	79% (230)	70% (393)	0.006^c^
Placebo	82% (241)	75% (411)	0.037^c^

^a^Patients receiving steroids at baseline or infusion were classified as receiving steroids. ^b^By analysis of variance. ^c^By Pearson's χ^2 ^test. ^d^Baseline shock was shock at any time within 6 hours prior to drotrecogin alfa (activated) or placebo infusion. APACHE, Acute Physiology and Chronic Health Evaluation.

## References

[B1] Hahn EO, Houser HB, Rammelkamp CH, Denny FW, Wannamaker LW (1951). Effect of cortisone on acute streptococcal infections and post streptococcal complications. J Clin Invest.

[B2] Schumer W (1976). Steroids in the treatment of clinical septic shock. Ann Surg.

[B3] Cronin L, Cook DJ, Carlet J, Heyland DK, King D, Lansang MA, Fisher CJ (1995). Corticosteroid treatment for sepsis: a critical appraisal and meta-analysis of the literature. Crit Care Med.

[B4] Lefering R, Neugebauer EA (1995). Steroid controversy in sepsis and septic shock: a meta-analysis. Crit Care Med.

[B5] Bennett IL, Finland M, Hamburger M, Kass EH, Lepper M, Waisbren BA (1963). The effectiveness of hydrocortisone in the management of severe infections. JAMA.

[B6] Klastersky J, Cappel R, Debusscher L (1971). Effectiveness of betamethasone in management of severe infections. A double-blind study. N Engl J Med.

[B7] Sprung CL, Caralis PV, Marcial EH, Pierce M, Gelbard MA, Long WM, Duncan RC, Tendler MD, Karpf M (1984). The effects of high-dose corticosteroids in patients with septic shock. A prospective, controlled study. N Engl J Med.

[B8] The Veterans Administration Systemic Sepsis Cooperative Study Group (1987). Effect of high-dose glucocorticoid therapy on mortality in patients with clinical signs of systemic sepsis. N Engl J Med.

[B9] Bone RG, Fisher CJ, Clemmer TP, Slotman GJ, Metz CA, Balk RA (1987). A controlled clinical trial of high-dose methylprednisolone in the treatment of severe sepsis and septic shock. N Engl J Med.

[B10] Luce JM, Montgomery AB, Marks JD, Turner J, Metz CA, Murray JF (1988). Ineffectiveness of high-dose methylprednisolone in preventing parenchymal lung injury and improving mortality in patients with septic shock. Am Rev Respir Dis.

[B11] Slusher T, Gbadero D, Howard C, Lewison L, Giroir B, Toro L, Levin D, Holt E, McCracken GH (1996). Randomized, placebo-controlled, double blinded trial of dexamethasone in African children with sepsis. Pediatr Infect Dis J.

[B12] Bollaert PE, Charpentier C, Levy B, Debouverie M, Audibert G, Larcan A (1998). Reversal of late septic shock with supraphysiologic doses of hydrocortisone. Crit Care Med.

[B13] Chawla K, Kupfer Y, Goldman I, Tessler S (1999). Hydrocortisone reverses refractory septic shock. Crit Care Med.

[B14] Briegel J, Forst H, Haller M, Schelling G, Kilger E, Kuprat G, Hemmer B, Hummel T, Lenhart A, Heyduck M (1999). Stress doses of hydrocortisone reverse hyperdynamic septic shock: a prospective, randomized, double-blind, single-center study. Crit Care Med.

[B15] Annane D, Sebille V, Charpentier C, Bollaert PE, Francois B, Korach JM, Capellier G, Cohen Y, Azoulay E, Troche G (2002). Effect of treatment with low doses of hydrocortisone and fludrocortisone on mortality in patients with septic shock. JAMA.

[B16] Minneci PC, Deans KJ, Banks SM, Eichacker PQ, Natanson C (2004). Meta-analysis: the effect of steroids on survival and shock during sepsis depends on the dose. Ann Intern Med.

[B17] Confalonieri M, Urbino R, Potena A, Piattella, Parigi P, Puccio G, Della Porta R, Giorgio C, Blasi F, Umberger R, Meduri GU (2005). Hydrocortisone infusion for severe community-acquired pneumonia: a preliminary randomized study. Am J Respir Crit Care Med.

[B18] Annane D, Bellissant E, Bollaert PE, Briegel J, Keh D, Kupfer Y (2004). Corticosteroids for severe sepsis and septic shock: a systematic review and meta-analysis. BMJ.

[B19] Keh D, Sprung CL (2004). Use of corticosteroid therapy in patients with sepsis and septic shock: an evidence-based review. Crit Care Med.

[B20] Burry LD, Wax RS (2004). Role of corticosteroids in septic shock. Ann Pharmacother.

[B21] Cooper MS, Stewart PM (2003). Corticosteroid insufficiency in acutely ill patients. N Engl J Med.

[B22] Annane D, Sebille V, Troche G, Raphael JC, Gajdos P, Bellissant E (2000). A 3-level prognostic classification in septic shock based on cortisol levels and cortisol response to corticotropin. JAMA.

[B23] Bernard GR, Vincent JL, Laterre PF, LaRosa SP, Dhainaut JF, Lopez-Rodriguez A, Steingrub JS, Garber GE, Helterbrand JD, Ely EW (2001). Efficacy and safety of recombinant human activated protein C for severe sepsis. N Engl J Med.

[B24] Dellinger RP, Carlet JM, Masur H, Gerlach H, Calandra T, Cohen J, Gea-Banacloche J, Keh D, Marshall JC, Parker MM (2004). Surviving Sepsis Campaign guidelines for management of severe sepsis and septic shock. Crit Care Med.

[B25] Hamrahian AH, Oseni TS, Arafah BM (2004). Measurements of serum free cortisol in critically ill patients. N Engl J Med.

